# Engineering a reporter cell line to mimic the high oligomannose presenting surface immunoglobulin of follicular lymphoma B cells

**DOI:** 10.1038/s41598-020-79862-2

**Published:** 2021-01-08

**Authors:** Butaek Lim, LeNaiya Kydd, Justyn Jaworski

**Affiliations:** Department of Bioengineering, University of Texas at Arlington, 500 UTA Blvd., Arlington, TX 76019 USA

**Keywords:** Tumour biomarkers, Lymphoma

## Abstract

Subtypes of B cell non-Hodgkin’s lymphomas, including follicular lymphomas, have shown a unique high oligomannose presentation on their immunoglobulins that will interact with natural receptors of the innate immunity, reportedly causing stimulation and proliferation. From deep sequencing of the variable heavy and light chain sequences of follicular lymphoma involved tissue sections, we identified the consensus variable sequences possessing glycosylation sites at the complementarity determining region. Using this information, we developed a cell line, referred to here as BZ, which displays the consensus variable segments as part of a surface antibody (IgM) and confirmed its presentation of high oligomannose on the heavy chain both in vitro and in vivo. An mCherry expressing variant provided a reporter cell line displaying the high oligomannose surface biomarker while affording clear fluorescent signals for FACS screening as well as for fluorescent in vivo imaging of ectopic xenograft tumors. In developing this reporter cell line that displays the biomarker glycan of follicular lymphoma, we provide a tool that may be used for future screening and validation of receptive moieties for selectively binding high oligomannose for development of targeted diagnostics or therapeutics to such B cell malignancies that display this unique glycan.

## Introduction

B cell follicular lymphoma as well as subsets of other B cell lymphomas have been found to display a unique high oligomannose glycan on their membrane bound immunoglobulin (Ig)^1–6^. Recent works examining the Ig variable regions of a large cohort of follicular lymphoma (FL) patients has shown an interesting common trait of presentation of a glycosylation site sequence within the variable segment, typically at the complementarity determining region (CDR)^4,7,8^. This site has been previously reported in the variable heavy chain of B-cell receptors of at least 90% of FL patients and at least 41% of Diffuse Large B-Cell Lymphoma (DLBCL) patients^1,2,4,8,9^, where such N-linked glycosylation sites of the CDR results in the unique display of a high oligomannose glycan. The displayed high oligomannose on the CDR may bind to receptors present on natural innate immune cells^3,10^. While N-glycosylation sites are known to be highly conserved within certain Ig heavy chain constant regions, the presence of N-glycosylation sites within the CDR are rare in healthy B-cells^10^. Endogenous oligomannose receptors, including DC-SIGN and DC-SIGNR, are presented by macrophage and dendritic cells within the germinal center, and these interactions with the oligomannosylated Ig of FL B-cells have been validated to result in B-cell receptor crosslinking for persistent activation of B cell proliferation^11,12^. The acquisition of this N-linked glycosylation motif is evident to be a required feature among certain B cell lymphomas. As somatic hypermutations of the antibody variable region persist in FL B cells producing a heterogeneous subclonal population, the conserved glycosylation sites at the CDR of follicular lymphoma B cells will remain; thus, glycosylation sites are retained in progression-associated subclones while glycosylation motif-negative subclones disappear from the overall tumor mass^9,11,13^. This conservation of the CDR glycosylation site during somatic hypermutation suggests it to be a critical factor that restricts dependence of FL on the surrounding tumor microenvironment^9^. It thus follows rationally why there continues to be great difficulty in generating a FL cell line or in vivo model given this dependence^3^.

While we now know that displayed oligomannose on the variable Ig domains can be bound by endogenous lectins of innate immune cells within lymphoid tissue, future efforts may look to identify mechanisms to block this B-cell receptor signaling required for FL B-cell survival. In looking to understand how this unique oligomannose can be presented as a feature on the CDR of Ig, it is important to consider that in the context of healthy mammalian systems these terminal mannose units of oligomannose are generally masked by other sugar moieties capped onto the end and thus oligomannose typically only exists as precursors, or core/internal sequences of more mature glycans. This biosynthesis of N-linked glycans begins through the transfer of an oligosaccharide composed of Glc_3_Man_9_GlcNAc_2_ to a nascent polypeptide chain in the endoplasmic reticulum at canonical glycosylation sites having the amino acid sequence (N-X-S/T). Reactions with glucosidases begin trimming the glucose terminal units while a series of mannosidases would follow to remove all but three mannose residues as the protein enters the early Golgi to follow with capping of terminal sugar residues such as fucose and sialic acid^14,15^. Natural recognition of high oligomannose at key checkpoint stages is indicative of improper folding whereby such oligomannosylated proteins would be targeted for degradation^16^. However, for an Ig of FL to display oligomannose without being targeted for degradation, it is believed that the commonly concave shape of the CDR^17^ may sterically block the glycan trimming enzymes as well as trafficking machinery from binding the oligomannose within the CDR pocket and in turn hinder the ability to degrade the high oligomannose displaying Ig. This distinct high oligomannosylation is consistently presented on the variable regions of the Ig in FL, while the constant domains possess properly processed glycosylation sites^12,15^. This feature provides a clear internal control that normal transit and processing through the Golgi is occurring but with an inability to process mannose trimming in the variable region due to some form of steric blocking as mentioned above. In order to provide a system for the display of the high oligomannose biomarker presented by the immunoglobulin of FL B cells, we have developed here a reporter cell-line which presents the variable domains from a FL B cell as a membrane bound surface antibody and validated its display of the high oligomannose glycan.

It has been reported that the availability of mouse models for examining FL are limited due to the difficulty to establish FL xenografts, as the local tumor microenvironment is required for their survival and immuno-compromised mice lack the necessary supportive interaction or mature secondary lymphoid organs^18^. Because of the requirement of this supportive cellular framework, in vitro growth has also shown very limited survival and as such there are said to be no true follicular lymphoma B-cell lines^18^. Nonetheless, a number of reports have demonstrated FL-like cell lines, often with co-culture of helper cells, which can serve as important tools for studying different aspects of FL such as chromosomal features, apoptosis, and cytokine-mediated growth regulation^19–24^. While molecular genetic and immunophenotypic features had been examined in the aforementioned studies, there were no reports of the glycosylation status of the surface immunoglobulin presented in these cell lines. In the following work we do not try to provide a FL cell line but rather provide an engineered reporter cell line that produces a high oligomannosylated surface antibody as to mimic the glycan target presented by FL B cells. While existing cell lines, such as the TKO pre-B cell line, have proven a valuable tool in understanding B cell development and signaling^25,26^, we have avoided the use of existing B cell lines as the intent is to develop a reporter cell line for use in receptor screening process. Thus, we instead selected the HEK293T cell line to ensure that surface features that are common to B cells would not be presented within our oligomannosylated surface IgM displaying reporter cell line. While other orthogonal cell lines could have been selected, the HEK293T cell line is one of the most widely used for expression and characterization of recombinant glycoproteins and is capable of a high degree of glycosylation complexity making it an amenable platform for cell-based glycan display^27^. Here, we provide our approach to using deep sequencing of variable domains from FL tissue sections to identify and generate a genetic construct for expression of a membrane bound IgM that displays the canonical high oligomannose on the heavy chain CDR. Herein, two engineered cell lines were produced using a HEK293 background and were found to stably express the oligomannoyslated antibody with one possessing an mCherry fusion in the cytoplasmic domain serving as a fluorescent reporter for the presence of the membrane-bound Ig. We anticipate providing these cell lines as tools for the research and development of receptive moieties for high oligomannose will help to yield new diagnostic or therapeutic technologies. The potential use of this reporter cell line for in vitro screening or in vivo imaging studies may serve as a starting point toward identifying relevant high oligomannose receptors that may eventually be advanced into clinically useful probes.

## Results

### Identifying established follicular lymphoma B cell antibody sequence possessing characteristic CDR glycosylation site

**Figure 1:**
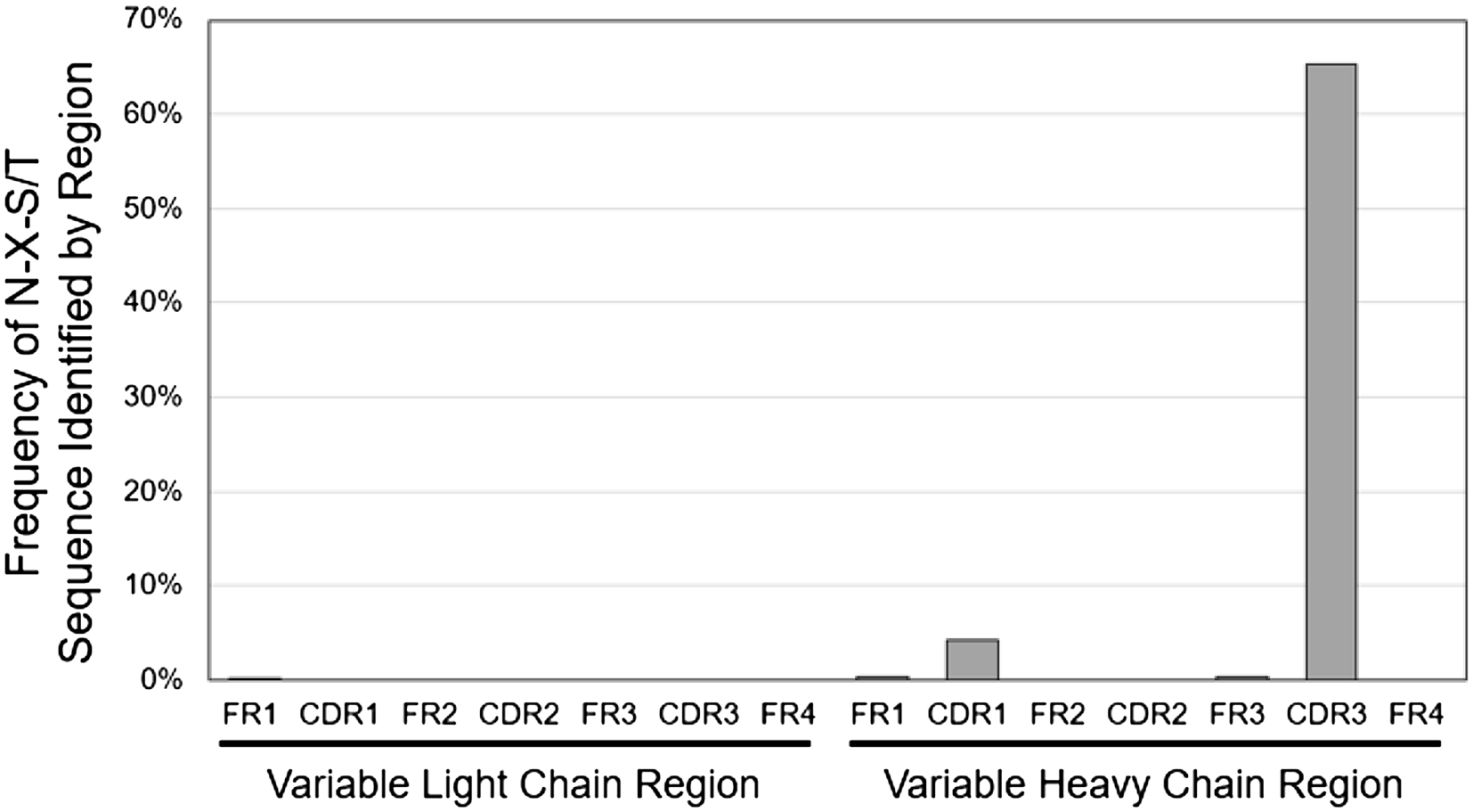
Frequency and location of glycosylation site sequences appearing in variable segments. Deep sequencing results of tissue sections from a follicular lymphoma involved lymph node were analyzed to reveal the region and frequency of total reads possessing an N-glycosylation site sequence N-X-S/T (where X is not Proline) suggesting that the B cells present in the section had preferential glycosylation of the CDR3 region.

Aberrant glycosylation sites on the (CDR) of the Ig presented by follicular lymphoma B cells have been widely reported as characteristic of this subset of lymphoma. To produce an engineered cell line which displays an established FL-derived antibody sequence, we conducted next generation deep sequencing (NGS) of the variable heavy and light chains presented from a FL involved lymph node tissue section. From NGS results, we identified 9931 unique sequence pairs (5228 unique to sample 1 among 216,053 paired-reads and 4797 unique to sample 2 among 155,596 paired-reads) of the variable domain amplicons. Examination of the amplicons by IMGT/HighV-QUEST revealed 3225 recombination to be productive which were distributed as 1798 heavy chain V-D-J recombinations, 1420 lambda light chain V-J recombinations, and only 7 kappa light chain V-J recombinations. While in normal reactive lymphoid populations there is a mixture of kappa versus lambda light chains, our observed lambda light chain dominance within the population was expected since light chain restrictions are a common feature of lymphomas^28^. Alignment and translation by IMGT/HighV-QUEST^29–32^ provided 260 unique variable heavy chain and 792 unique variable light chain domain among which 174 heavy chains and 1 light chain possessed the consensus N-X-S/T glycosylation sequence. As proline creates too much distance between the acceptor asparagine from that of the hydroxyl of serine or threonine for glycosylation machinery, N-linked glycosylation does not typically proceed when the X residue is a proline and as such those sequences possessing an N-P-S/T sequence were not considered glycosylation sequences in our analysis^33^. From Fig. 1, we can see the distribution of these glycosylation sequences were predominantly at CDR3 and to a lesser extent at CDR1 of the heavy chain. This agrees with recent reports of aberrant glycosylation sites found on the antibodies of follicular lymphoma B cells^9^.

Multiple sequence alignment using MAFFT (multiple alignment using fast Fourier transform) was implemented for the heavy chain and lambda light chain variable domains and the sequence pattern of the clones present in the follicular lymphoma B cells are presented in Supplementary Figure S7 as a sequence logo. The consensus heavy chain clonotype was found to be derived from the IGH V3–23 germline while that of the lambda light chains were derived from IGL V1–47. Importantly, an N-D-S glycosylation site at the heavy chain CDR3 was highly conserved, where reports have shown such aberrant glycosylation sites at the CDR to be characteristic of FL.

The dominant variable heavy chain and light chain found from deep sequencing of the single follicular lymphoma involved lymph node were thus identified. The heavy chain variable domain consensus sequence (E-V-Q-L-L-E-S-G-G-G-L-V-Q-P-G-G-S-L-R-L-S-C-A-A-S-G-F-T-F-N-T-F-S-M-N-W-V-R-Q-G-P-G-Q-G-L-D-W-V-A-G-I-S-G-S-G-T-I-T-Y-Y-A-D-S-V-R-G-R-F-S-I-S-R-D-N-S-K-N-T-L-Y-L-E-M-K-S-L-R-V-E-D-T-A-V-Y-Y-C-A-N-**N-D-S**-S-G-P-I-Y-F-E-S-W-G-Q-G-T-L-V-T-V-S-S) possessing the glycosylation site and the CDR3 and the lambda light chain variable domain consensus sequence (Q-S-V-L-T-Q-P-P-S-A-S-G-T-P-G-Q-R-V-T-I-S-C-S-G-S-S-S-N-I-G-S-N-Y-V-Y-W-F-Q-Q-L-P-G-S-A-P-K-L-L-I-Y-R-D-S-Q-R-P-S-G-V-P-D-R-F-S-G-S-K-S-G-T-S-A-S-L-A-I-S-G-L-R-S-E-D-E-A-D-Y-Y-C-A-A-W-D-D-S-L-S-G-R-V-F-G-T-G-T-Q-L–T-V-L) were found to be in agreement among the clonotypes having among the most abundant reads for the heavy and light chains variable domains. The above sequences were used to construct an antibody expression system to display the FL B cell derived variable domains on the membrane bound surface IgM for our engineered cell line.

### Confirmation of engineered cell line stably expressing surface IgM derived from FL B cell

**Figure 2:**
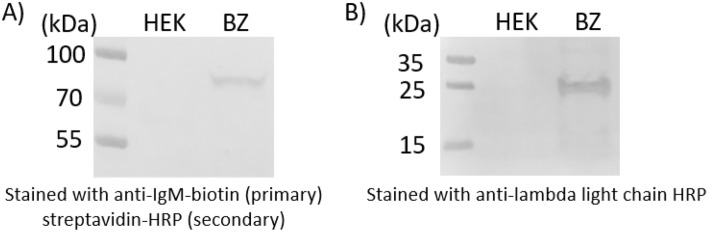
Immunoblotting of the engineered BZ and HEK background cell lysates separated by SDS-PAGE. (**A**) Heavy chain anti-IgM staining and (**B**) light chain anti-lambda staining of the cell lysates from the engineered BZ cell line and the HEK background from which BZ was derived reveal expression of IgM heavy chain and lambda light chain for the BZ cell line but not observed for the HEK background. The light chain band appears at ~ 25 kDa which is the expected size for the unglycoyslated light chain, while the heavy chain appears larger than the unglycosylated molecular weight of ~ 66 kDa indicating that the heavy chain has indeed been glycosylated.

After construction of the expression vectors for the IgM heavy chain possessing a transmembrane domain and for the Ig lambda light chain, the transfection of HEK293T cells followed by antibiotic selection. The use of antibiotics (blasticidin and zeocin) above the kill curve threshold for HEK293 were used for selection of clones facilitating the successful isolation of an engineered cell line (which we refer to as the BZ cell line) that provided stable expression of surface IgM. Confirmation of the heavy chain and light chain expression was confirmed by western blot as seen in Fig. 2.

The expected size of the IgM heavy chain possessing a transmembrane domain and the FL B cell derived variable domain consensus sequence was expected to have an unglycosylated molecular weight of ~ 66 kDa while the lambda light chain was expected to have an unglycosylated molecular weight of ~ 25 kDa. A band size for the heavy chain larger than 66 kDa was seen by immunoblotting indicating the IgM heavy chain was indeed glycosylated. In contrast, immunoblotting revealed the expected size for an unglycosylated light chain. The anti-IgM and anti-lambda stained bands were observable only for the case of the engineered BZ cell lysate and not for the HEK lysate control. This shows that the HEK background did not previously possess any cross-reactive proteins, and further confirms the expression of the full length heavy chain and lambda light chain.

### Examination for oligomannosylation of antibodies presented by the engineered BZ cells

**Figure 3:**
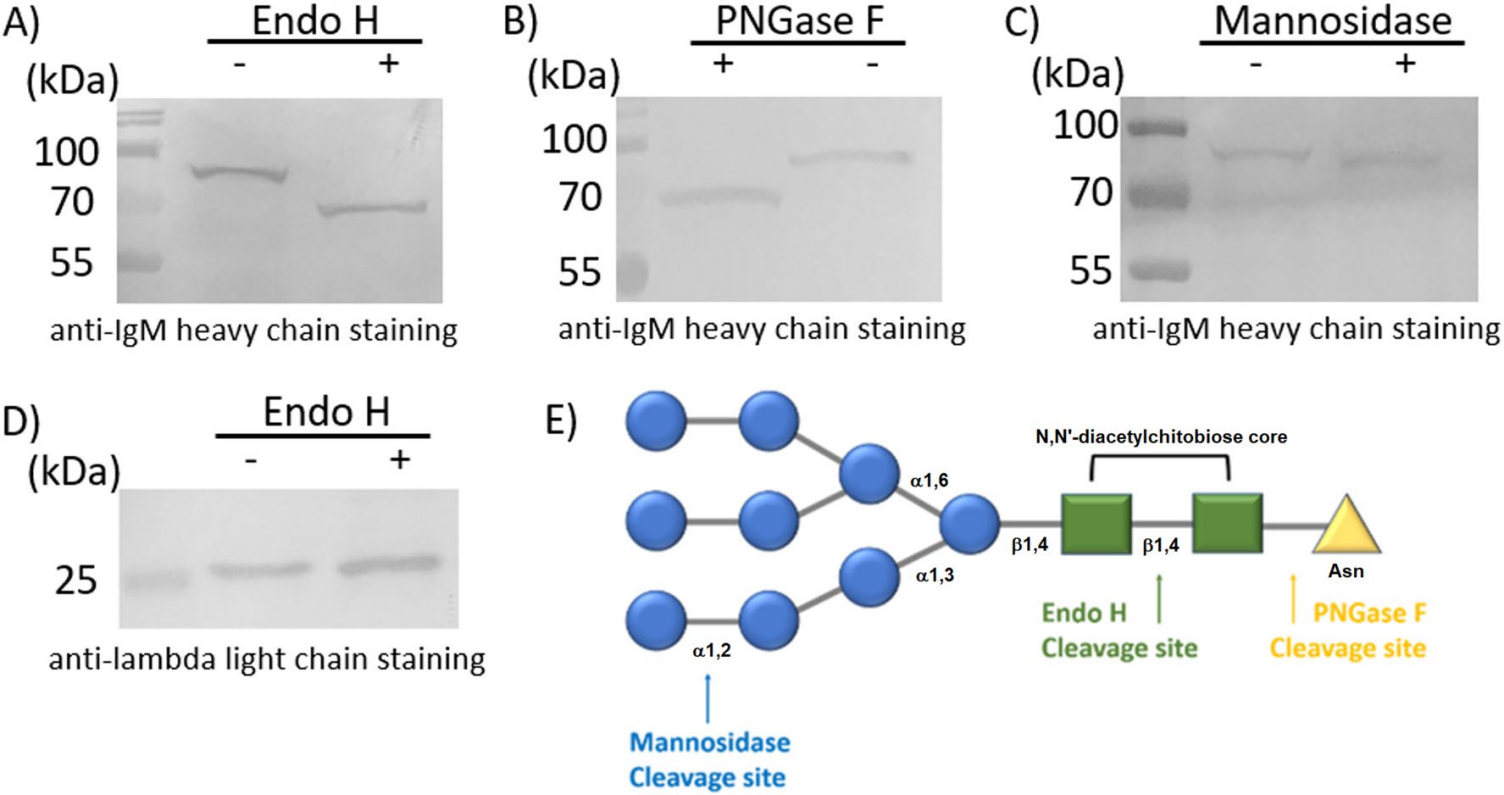
Immunoblotting of glycosidase assay product. Cell lysates from engineered BZ cells were used for glycosidase assays using (**A**,**D**) EndoH, (**B**) PNGase F, or (**C**) Mannosidase and separated by SDS-PAGE. Immunoblotting of the transfers and staining with (**A**–**C**) anti-IgM heavy chain (primary) or (**D**) anti-lambda light chain (primary) revealed a shift in the size of the heavy chain resulting from cleavage of the glycan while the light chain size was unchanged. A smaller band shift for cleavage by Mannosidase relative to EndoH or PNGase F is expected based on (**E**) the location of the cleavage sites.

Here we examined if the glycosylation site on antibody presented by our BZ cell line would result in the display of aberrant high oligomannose as is seen on the antibodies of follicular lymphoma B cells. To do so, we conducted a series of high mannose specific glycosidase assays followed by western blot staining for the heavy and light chains. As shown in Fig. 3, the assays using the BZ lysate revealed the appearance of different band shifts relative to the untreated BZ lysate. This shift corresponds to the cutting away of glycans by the distinct glycosidases implemented in the assays. The specificity of the EndoH and Mannosidase enzymes in digesting only glycans terminating in high oligomannose allowed our verification by immunoblotting that high oligomannose was indeed presented by the heavy chain as observable by the band shifts. In contrast, the light chain revealed no band shift suggesting no high oligomannosylation. This was to be expected given our knowledge of the location of the glycosylation site at the CDR of the heavy chain. No such glycosylation site was present in the light chain of the engineered BZ cell line and this is in agreement with the immunoblotting results as either with or without EndoH glycosidase treatment the light chain band remain at the expected molecular weight of 25 kDa. In contrast, a predicted decrease in the molecular weight of the heavy chain was observed based on the specificity of the glycosidase to which it was exposed. For example, the EndoH cleaves almost the entirety of high mannose glycan by cutting at the chitobiose core, thus showing a large decrease in the molecular weight of the IgM toward the expected unglycoyslated size of ~ 66 kDa. PNGase will cleave the entire glycan, and it recognizes and cleaves between the innermost GlcNAc and asparagine residues of all N-linked glycoproteins and not only high mannose glycans. As such PNGase F treatment showed the IgM size to again decrease to ~ 66 kDa indicating the release of the N-linked glycan. In contrast, Mannosidase trims only the terminal mannose residues within high oligomannose and thus displayed a much smaller decrease in the molecular weight of the IgM as compared to the other glycosidases. The results confirmed that the engineered BZ cell line could display surface IgM with the high oligomannose glycosylation as is presented by follicular lymphoma B cells from which the variable segments had originated. Additional confirmation of the high oligomannosylation of our reporter cell line was carried out using a lectin-based binding assay (Supplementary Figure S8) in which DC-SIGN and DC-SIGNR labeled magnetic beads were found to provide enrichment for our reporter cell line in mixed cell samples. In addition, a negative control cell line was produced which possesses a mutated version of the IgM heavy chain that lacks the N-D-S glycosylation site of the CDR3 and may serve as a useful negative selection target for removing high oligomannose target-unrelated clones in future screening assays. As seen in Supplementary Figure S9, the IgM heavy chain of the negative control cell line has a decreased size owing to glycan loss at the CDR3 as compared to that displayed by the BZ cell line.

### Developing murine tumors from engineered BZ cell line

**Figure 4:**
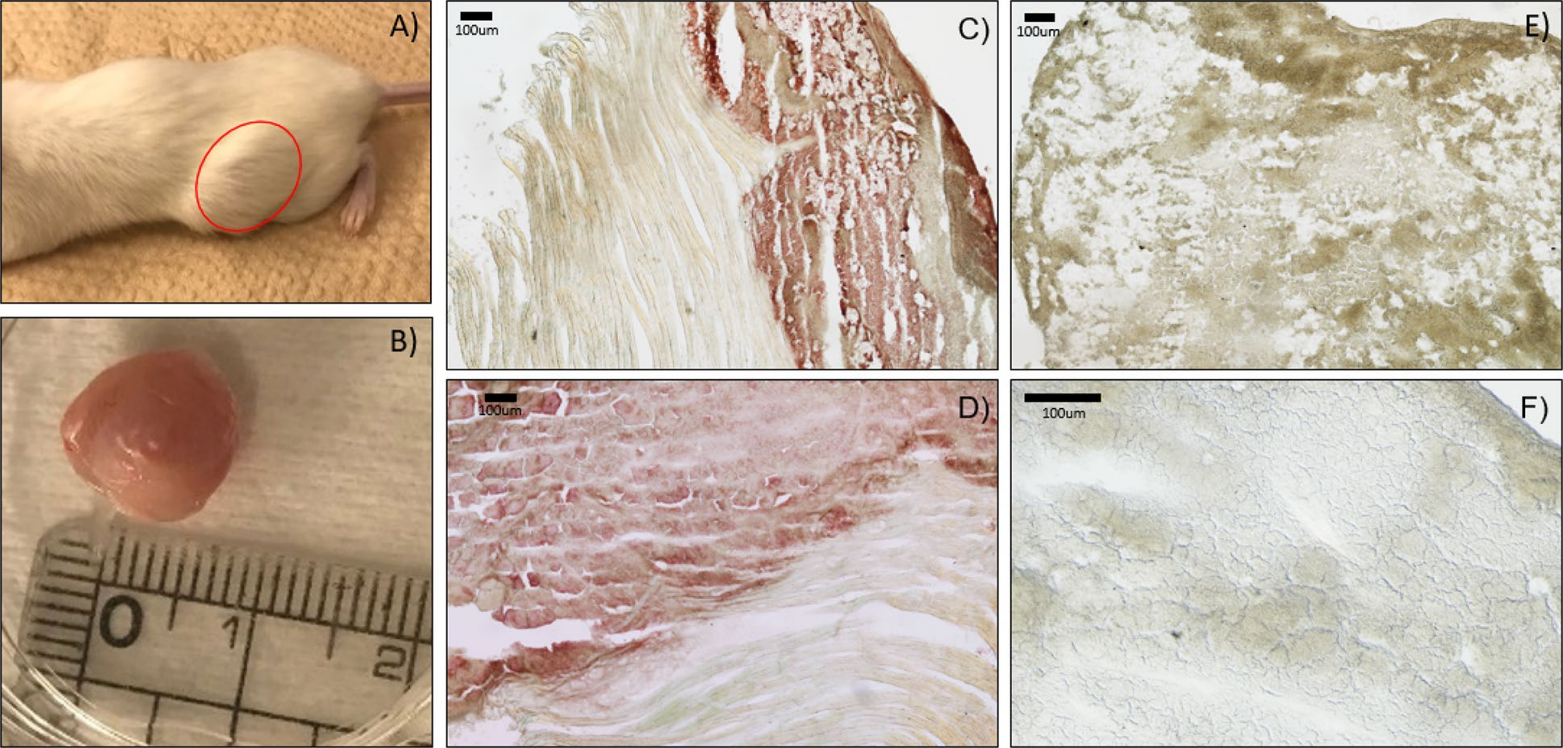
Tumor engraftment and immunohistochemistry. (**A**) Palpable tumors formed at 4–5 weeks after subcutaneous injection of BZ cells into the flank of NOD-scid mice were (**B**) removed and (**C**,**D**) cryosectioned for immunohistochemistry. (**E**,**F**) HEK cell derived tumor were similarly grown, removed and section. Positive IHC staining of BZ tumor sections was observed with (**C**) anti-lambda light chain and (**D**) anti-IgM heavy chain at the location of the tumor but not stained in the adjacent muscle tissue. In contrast, HEK derived tumors exhibited a negative signal for (**E**) anti-lambda light chain and (**F**) anti-IgM heavy chain IHC staining.

To examine if tumors developed from the engineered BZ cell line could be engrafted and still present the surface IgM, we generated ectopic murine tumors in NOD-SCID mice using the BZ cell line as compared to the HEK293 background. We confirmed the appearance of tumors after 4–5 weeks with an approximate size of 9 mm. Tumors were excised and were subjected to either lysis for analysis by glycosidase assay and western blot or flash frozen in OCT for cryosectioning. From Fig. 4, we see that IHC staining revealed the positive presence of heavy and light chain for the engineered BZ tumor sections but was not seen for the control HEK tumor sections which confirms in vivo antibody expression by BZ derived tumors. Furthermore, glycosidase assays specific for high oligomannose conducted for tumor lysate samples confirmed high oligomannose display on the IgM heavy chain of BZ tumors but not HEK tumors (Supplementary Figure S1).

### Examining BZ-mCherry reporter cell line

**Figure 5:**
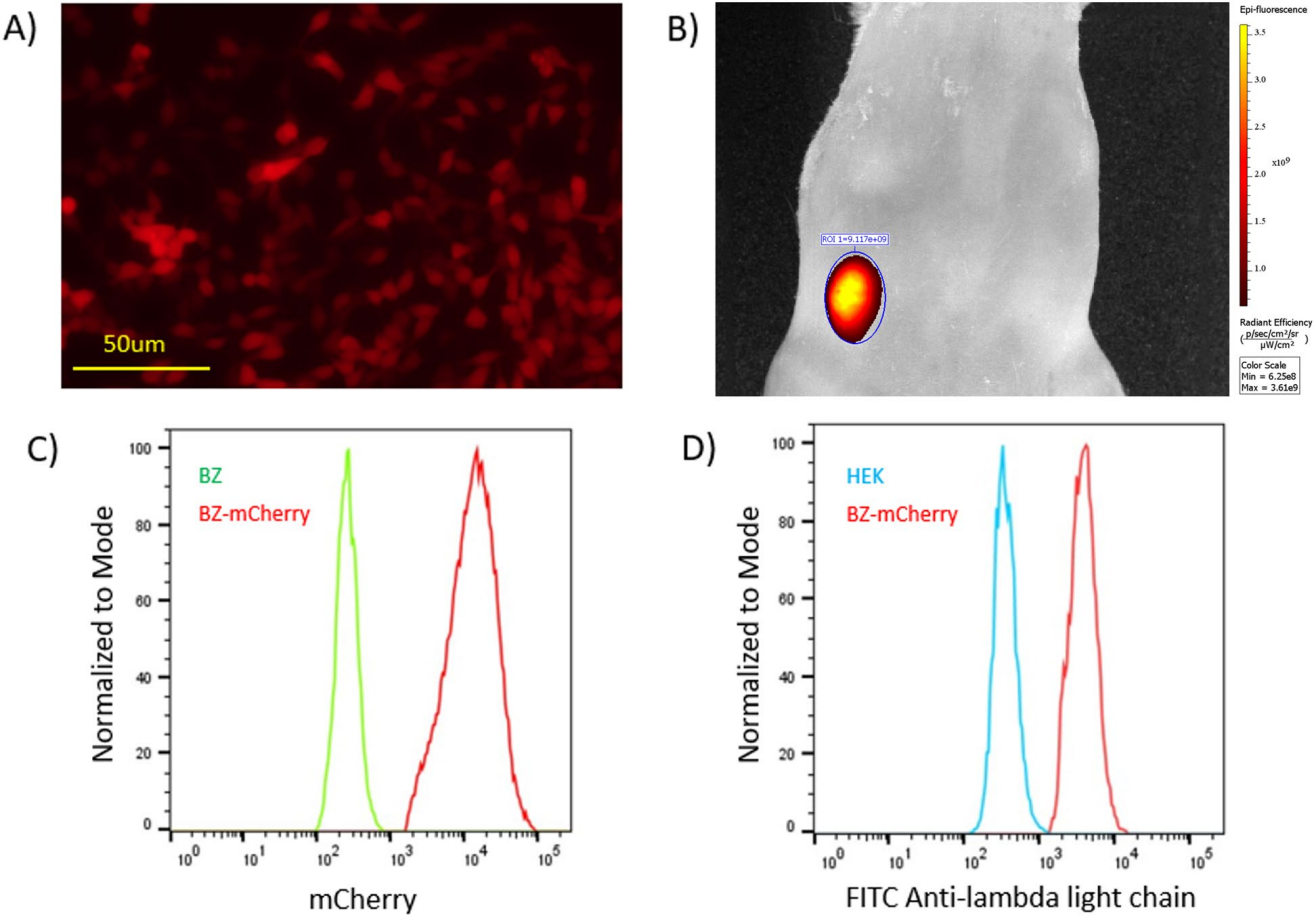
FACS and fluorescence in vivo imaging. (**A**) Fluorescent microscopy of engineered BZ-mCherry cell line shows the clear red fluorescence of the reporter cell line (scale bar = 50um). (**B**) Ectopic murine BZ-mCherrry tumors are clearly visible by fluorescence in vivo imaging taken at Em = 620, Ex = 580, Bin = 4/4, Fnumber = f2, exposure = 0.5 s. (**C**) The potential use of the BZ-mCherry cell line for FACS based screening is also evident by the bright red fluorescence. (**D**) We also see a large shift in the mean fluorescent intensity for the BZ-mCherry cells compared to HEK when incubated with FITC-labeled anti-lambda probe.

The reporter cell line BZ-mCherry (possessing a cytosolic fluorescent mCherry fusion to the IgM heavy chain) was examined for its potential to be used as a tool for some of the more common in vitro and in vivo targeting techniques of FACS and fluorescence in vivo imaging, respectively. It is important to note that the fluorescent mCherry signal as a fusion of the IgM does not directly report oligomannose presence but rather reports the heavy chain expression. From Fig. 5, the BZ-mCherry cell line is seen to provide a bright red fluorescence that is a readily observable by fluorescence microscopy, and tumors generated from BZ-mCherry are clearly visible with fluorescence in vivo imaging systems. The reporter BZ-mCherry cell line also holds the potential for being implemented in FACS studies as seen in Fig. 5C by the high mean fluorescence intensity in the red channel relative to the original BZ cell line. We also find when incubating the BZ, BZ-mCherry, and HEK cell lines with FITC-labeled anti-lambda light chain probe a clear increase in mean fluorescence intensity (MFI) was observable from FACS analysis (Fig. 5D) for the BZ-mCherry relative to the HEK control indicating the display of surface antibody, where this can similarly be seen (Supplementary Figure S2) for the BZ cell line. The ability for the reporter cell line to be readily distinguished from the non-reporter BZ cell line and HEK293 background cells by the red fluorescence by FACS suggest it to have potential utility for screening studies. Moreover, the ability to distinguish the reporter BZ-mCherry cells within an in vivo context when conducting fluorescent in vivo imaging of ectopic murine BZ-mCherry tumors suggests that this engineered reporter cell line may prove useful owing to its bright red fluorescence and display of the oligomannosylated antibody.

## Discussion

Recent works have shown that glycosylation of the Ig of follicular lymphoma B cells results in presentation of oligomannose on the CDR that is a necessary characteristic for their survival, as this glycosylation facilitates continual stimulatory interactions via the endogenous lectins in the surrounding tumor microenvironment^3,4^. Because of this dependency, it has been historically difficult to examine isolated patient derived FL cells in vitro for any extended period of time or arrive at a realistic FL cell line^18,19^. Here we explored the development of a cell line that reports expression of a membrane-bound Ig derived from follicular lymphoma B cell where this Ig displays a characteristic high oligomannose glycan as presented by follicular lymphoma B cells. As no oligomannose specific technologies have yet been able to target this biomarker, the intention of this work is to provide a possible tool for future development of suitable targeting moieties that could selectively bind the displayed oligomannose in order to facilitate targeted diagnostics or therapeutics that invoke disruption of the aforementioned lectin-oligomannose interactions. In this work, we first carried out deep sequencing of the Ig variable regions of FL involved lymph node sections, where we identified a conserved glycosylation site presented at the third complementarity determining region (CDR3) of the variable heavy chain. The consensus heavy chain possessed significant drift from the germline unlike other sequences found within the node indicating it had undergone significant somatic hypermutation. The sample was also found to be lambda light chain restricted with a clear predominance for a consensus lambda sequence. Using the consensus sequence from the follicular lymphoma B cell sample for the heavy and light chain variable segments, we genetically engineered a set of mammalian expression vectors incorporating the identified variable heavy and light chain sequences within a surface IgM and lambda IgL construct, respectively. Blasticidin and zeocin resistance within the heavy and light chain expression constructs allowed antibiotic selection of the transfected HEK cells and after continual growth we isolated cell lines with stably integrated heavy and light chain constructs. The cell line was found to be very stable with no loss of expression or display of the IgM heavy chain or lambda light chain. Examination of the glycosylation presented by the cell line was carried out using a series of glycosidase assays. Specifically, the use of Endoglycosidase H (Endo H) which cleaves the internal GlcNAc core of high mannose structures and the use of Mannosidase which trims only external mannose residues was implemented in order to confirm the presence of high oligomannose terminating glycans presented by the antibody on the engineered BZ cell line. We identified no oligomannose display by the lambda light chain, which was expected given the lack of a glycosylation site at the variable lambda CDRs. In contrast, we did find oligomannose to be displayed on the IgM which is expected given the glycosylation sequences found at the CDRs of the FL B cell derived variable heavy chain. From our glycosidase assays, we confirmed the presence of high oligomannose. The Endo H enzyme selectively cleaves high mannose terminating glycans, and immunoblotting revealed a large decrease in the size of the IgM after Endo H treatment. A slightly larger size shift was revealed for the use of PNGase F which is not high mannose specific but will more broadly cleave almost all N linked glycans of the high mannose type, hybrid type, and complex type. The fully deglycosylated heavy chain after PNGase F was seen to be in direct agreement with the expected unglycosylated molecular weight. The display of high oligomannose by the heavy chain was further validated by use of a third glycosidase assay using Mannosidase which has exoglycosidase specificity for terminal mannose residues. It is important to see that the Mannosidase treatment resulted in a significantly smaller decrease in the size of the heavy chain band as compared to the EndoH treatment, where this small shift was representative of the trimming of only the external mannose residues of high oligomannose present on the IgM. The fact there is a decreased size after treatment alone confirms the presence of the high oligomannose, but more importantly the smaller size shift relative to the band size found after cutting the entire glycan at the core GalNAc residues with EndoH and PNGase F suggests that the Mannosidase was specific in trimming the mannose from the high oligomannosylated IgM.

Given the successful display of the oligomannose biomarker on the surface IgM of our engineered cell line in vitro, we then moved to examining the cells in vivo using NOD-scid mice. We identified that our engineered BZ cell line when injected subcutaneously into the flanks of the mice at 5 × 10^6^ cells per 100 μL PBS could result in palpable tumor formation after 4–5 weeks. Excision of the tumors and immunohistochemical staining with anti-lambda and anti-IgM confirmed the presence of the surface antibody display throughout the tumor mass. The western blot analysis of the lysed tumor tissue also revealed the presence of the oligomannose was retained on the heavy chain as confirmed by endoglycosidase assay suggesting that the glycan display was not affected by the in vivo growth environment. We next looked to further developing a second cell line which possesses an additional component of an internal mCherry fluorescence reporter fused to the cytoplasmic side of the membrane bound IgM. The utility of this BZ-mCherry cell lines as a possible tool for screening of moieties that can bind the target high oligomannose may be extremely valuable in future therapeutics development and pre-clinical screening. One observed feature of the BZ cells relative to the HEK background was a decrease in the adhesion capabilities of the BZ and BZ-mCherry cells as compared to the HEK293 background from which they were derived for in vitro culture. This may be attributed to the display of surface Ig presented by the engineered BZ and BZ-mCherry cell lines in some manner restricting interaction of integrins with the surface but was not explored as it was out of the scope of this study. The HEK, BZ, and BZ-mCherry cell lines were also used for generating ectopic xenograft tumors in NOD-scid mice where it was shown that the oligomannosylation of the heavy chain was retained during in vivo growth for the BZ and BZ-mCherry tumor lysates.

In summary, we offer our development of an engineered fluorescent reporter cell line that provides the stable expression of membrane bound Ig that display high oligomannose on the CDR, as the Ig variable segments were derived from a follicular lymphoma B cell. Beginning from FFPE tissue slides of a lymph node with involved FL, we identified the dominant subclones presenting glycosylation sites on the CDR by deep sequencing, and we cloned the variable domains into an expression vector where the stable expression of surface antibody was confirmed in vitro and in vivo. Immunoblotting following glycosidase assays validated the oligomannosylation of the heavy chain, and a reporter cell line was then developed as a potential future screening tool. Here, the display of oligomannosylated surface Ig may serve as a mimetic target for in vitro or in vivo development and testing of high oligomannose binding moieties, which are not yet available for targeting to follicular lymphoma B cells. Unlike previous reports that have sought to create FL cell lines for in vitro studies, our approach does not try to incorporate the cell signaling features of follicular lymphoma B cells but instead the BZ cell lines strictly provides a key mimetic surface feature of the oligomannosylated antibody. We anticipate that this cell line may be used for future identification of a receptive moiety that can properly bind to the unique high oligomannose target presented by the Ig of certain B cell lymphomas. Because this target represents a critical element for not only diagnostic but also potential therapeutic purposes, we provide this as a starting point to open the potential for future work in developing clinically relevant technologies that may bind to high oligomannose.

## Methods

### Cloning and isolating cells that stably express consensus variable Ig domains on surface IgM

**Figure 6:**
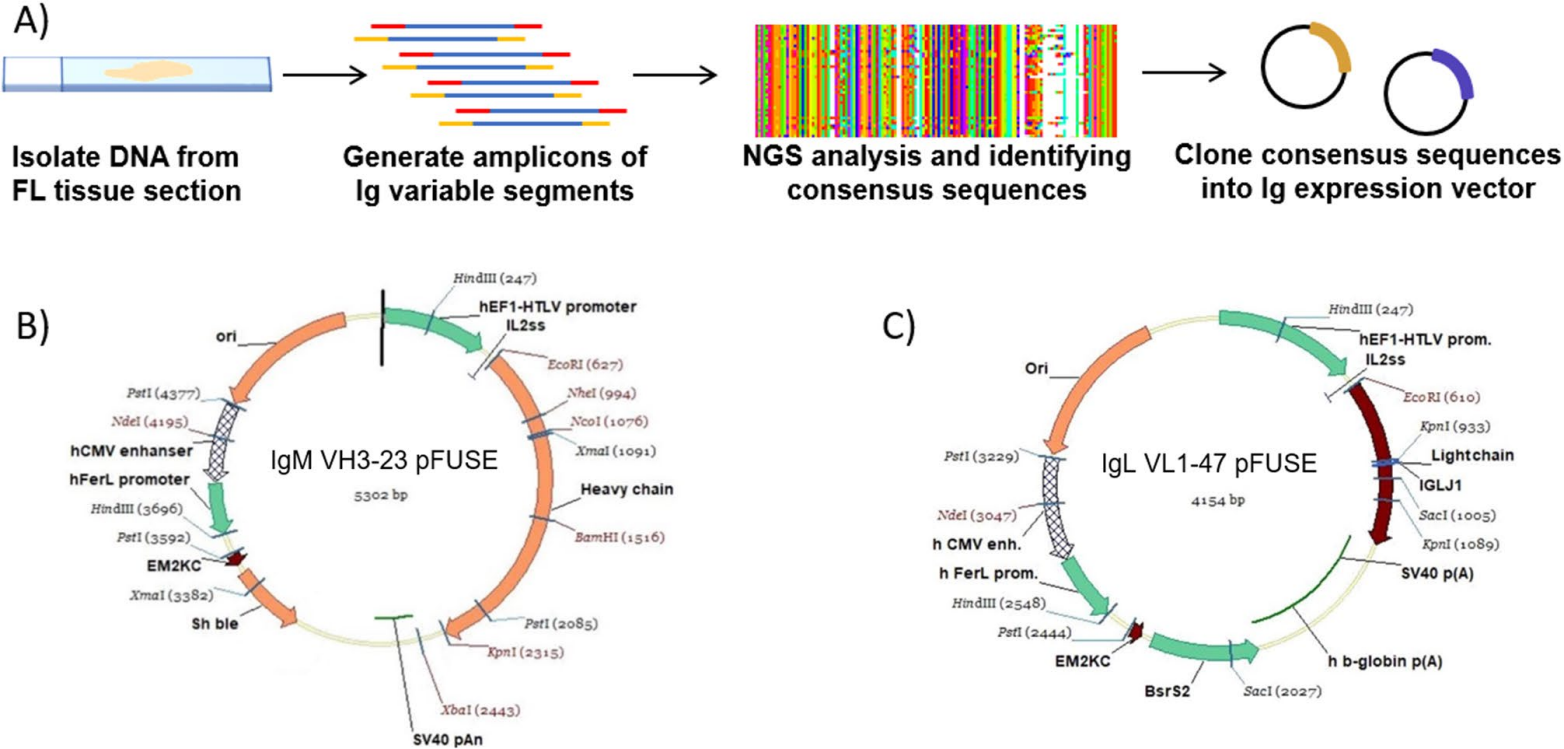
Process to generate Ig expression vectors possessing variable domains from follicular lymphoma B cells. (**A**) Schematic of our approach to identify consensus variable segment sequences from follicular lymphoma B cell tissue sections. (**B**) Plasmid map of IgM VH3-23 pFUSE vector and (**C**) IgL VL1-47 pFUSE vector used for production of surface antibody displaying the variable heavy and light chain consensus segments identified from FL B cell sections and carrying genes for zeocin and blasticidin resistance, respectively. Plasmid maps made using VectorNTI version 10.3.0 (Thermo Fisher Scientific, USA, https://www.thermofisher.com).

After identifying the dominant consensus sequences of the variable heavy and variable lambda light domains from the FL involved lymph node derived from the left groin of a 35 year old female, these variable heavy and light sequences were cloned into two modified pFUSE mammalian expression vector constructed as describe in detail in the Supplementary Methods and summarized in Fig. 6. Two different pFUSE expression vectors were used, one for expression of the heavy chain IgM containing zeocin resistance and one for expression of the lambda light chain containing blasticidin resistance.

**Figure 7:**
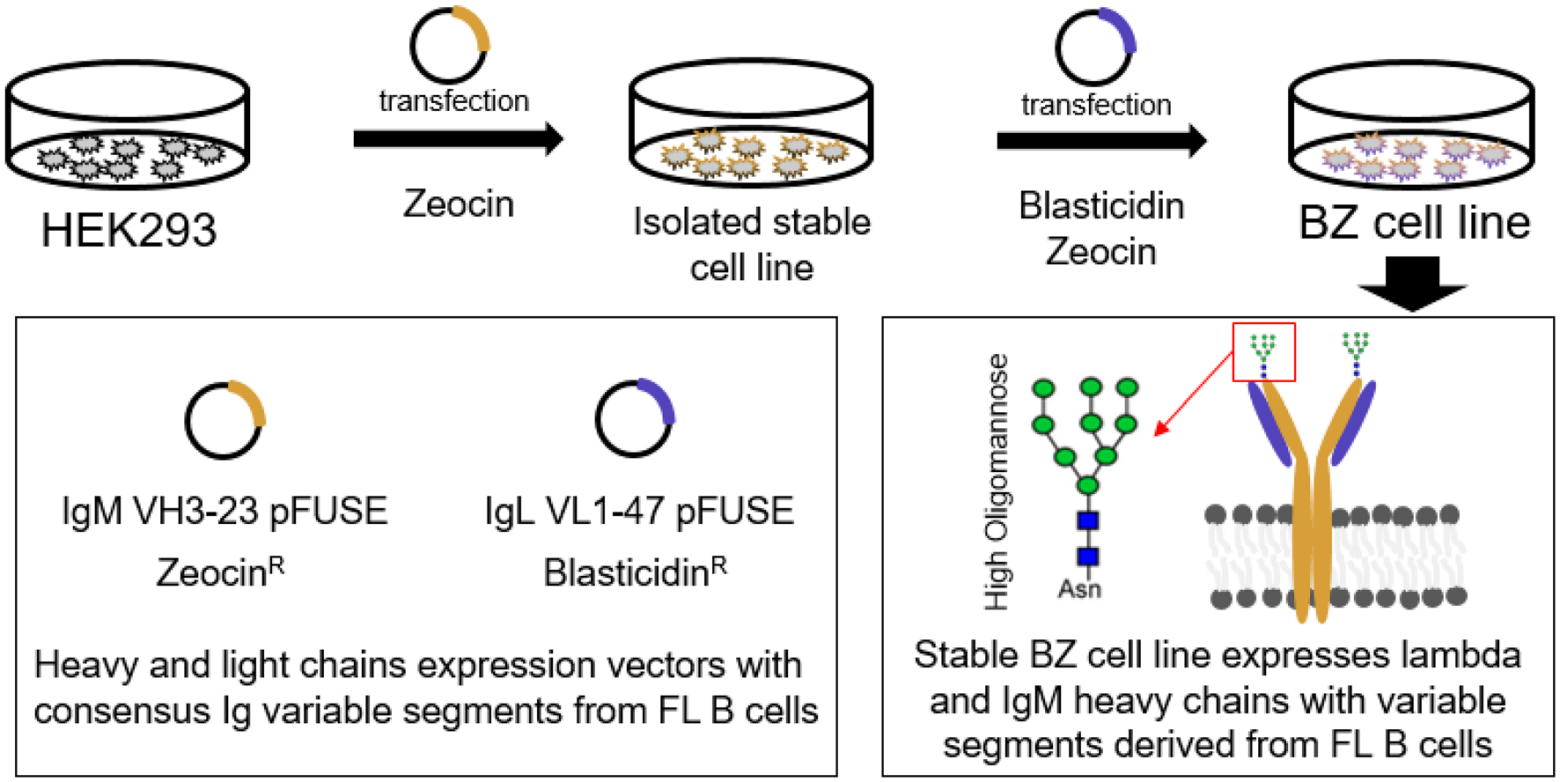
Schematic of process for generating engineered BZ cell line. (**A**) Expression vectors based of pFUSE were constructed with variable segments derived from consensus deep sequencing results of follicular lymphoma B cells. Sequential isolation of stable cell lines under antibiotic selection and examination of expression were carried out to identify the BZ cell line which provided the surface display of antibody possessing the FL B cell derived variable domains.

The IgM VH3-23 pFUSE was engineered to possess a canonical IgM transmembrane domain as to present the B cell receptor on the cell surface when the heavy and light chain pair are co-expressed. Co-transfection of 50% confluent HEK293T cells with the IgM VH3-23 pFUSE and IgL VL1-47 pFUSE plasmid was carried out in sequential transfection and selection step utilizing polyethyleneimine as carrier in 24 well plates. An overview of the process is provided in Fig. 7. HEK293T cells were chosen for creating an orthogonal reporter cell line as to eliminate the presence of intrinsic surface features displayed by B cells that may confound future screening efforts. This includes associated features such as CD79a/b which when eliminated have been known to reduce surface Ig expression in HEK293T cells^34^ among other cell types^35–37^. Incorporating a cytoplasmic heavy chain fusion however has shown when CD79a/b are absent that extending the cytoplasmic domain can enhances Ig surface display by up to 10,000 fold^38^. For the selection of stable transfectants with IgM VH3-23 and IgL VL1-47 integrated into the genome, zeocin and blasticidin were used respectively. The antibiotic was added to the culture media as selection markers beginning from 48 h after transfection and were allowed to grow for 4 days until colonies were isolated for subsequent screening. Screening of stable transfectants possessing the heavy and light chain after 3 weeks were carried out by separation on 96 well plates and examining replicate plates by IHC staining with HRP conjugated anti-Lambda Light Chain (Cat# NB7552 Novus Biologicals) and anti-IgM Heavy Chain (Cat# 1020-05 Southern Biotech Inc) antibody. The isolated cell lines possessing both blasticidin and zeocin resistance that were confirmed to display IgLambda and IgM were passaged to provide sufficient cells for examination of the lysate by western blot (as seen in Fig. 2). In brief, after SDS-PAGE of the cell lysate and overnight transfer to nitrocellulose membranes, the membranes were blocked in 1%BSA in PBS buffer overnight at 4 °C. For light chain examination, the membrane was incubated for 1 h in 10 mL of washing buffer containing 1uL of 1 mg/mL anti-Lambda Light Chain directly conjugated with HRP (Cat# NB7552 Novus Biologicals). For heavy chain examination, the membrane was incubated for 1 h with 10 mL of washing buffer containing 1 μL of 1 mg/mL biotinylated anti-IgM Heavy Chain (Cat# M31515 Invitrogen) followed by washing and addition of streptavidin conjugated HRP (Cat# 18-152 Sigma Aldrich). AEC staining was used to resolve the bands to confirm the expressed heavy and light chains on the nitrocellulose membrane. The BZ-mCherry cell line was established by the above method as well but with automated screening by FACS (BD FACS Melody) and visual assessment by fluorescence microscopy (Nikon ECLIPSE Ti2-A).

### Validation of oligomannosylation of IgM displayed on engineered cell line

To determine the presence of oligomannosylation on the heavy chain or light chain, glycosidases Endo H, PNGase F, or Mannosidase were added to the lysate of BZ the cell line and compared by western blot to lysate of the HEK293 background cell. Approximately, 10^6^ cells were lysed with 100 μL of RIPA buffer on ice for 30 min and centrifuged for 10 min at 13,000 rpm to pellet cell debris, and the supernatant was collected for further experiments. Enzyme reaction conditions were prepared by the supplier’s (New England Biolabs, MA, USA) instructions and their provided buffers. Briefly, mannosidase reaction was prepared by combining 8 μL of the above-mentioned cell lysate, 1 μL of 10 × GlycoBuffer 4 (1 × is 50 mM sodium acetate pH 4.5) and 1 μL of Mannosidase, and the mixture was incubated at 37 °C for 1 h. For Endo H and PNGase F enzyme reaction, the cell lysate was denatured by combining 9 μL of cell lysate and 1 μL of 10 × denaturing buffer (1 × is 0.5% SDS and 40 mM DTT) and boiled at 100 °C for 10 min. 8 μL of denatured cell lysate, 1 μL of 10 × Glycobuffer 3 (1 × is 50 mM sodium acetate pH 6.0) and 1 μL of Endo H were combined and incubated for 1 h at 37 °C. For PNGase F reaction, 7 μL of denatured cell lysate, 1 μL of 10 × Glycobuffer 2 (1 × is 50 mM sodium phosphate pH 7.5), 1 μL of 10% NP-40 and 1 μL of PNGase F were mixed and incubated at 37 °C for 1 h. Each reactant was mixed with protein loading dye and boiled at 100 °C for 10 min. Samples were loaded and resolved by SDS-PAGE for 2 h at 120 V followed by overnight transfer to nitrocellulose membrane at 4 °C. Membranes were blocked in 1% bovine serum albumin (BSA) in 10 mM PBS buffer. For the images in Fig. 3, 1 μL of 1 mg/mL antibody, anti-IgM Heavy Chain conjugated with HRP (Cat# 1020-05 Southern Biotech Inc) or anti-Lambda Light Chain conjugated with HRP (Cat# NB7552 Novus Biologicals), was diluted with 1 mL of washing buffer (1% BSA and 0.5% Tween-20 in 10 mM PBS) and incubated overnight with slow horizontal agitation (50 rpm). After removal of the antibody containing buffer, the membrane was washed three times with 10 mL of washing buffer for 10 min each with horizontal agitation. 10 mL of 0.05% AEC and 0.015% H_2_O_2_ in 50 mM acetate buffer pH 5.5 was added to develop the membrane. The complete panel of immunoblots images are provide in Supplementary Figure S6.

### Flow assisted cell sorting

FACS analysis of the HEK293, BZ, and BZ-mCherry cells was performed using a BD FACS Melody system. Prior to analysis, cells were fixed and stored in 1% BSA in PBS. FITC labeled anti-lambda light chain (Cat# NB7551 Novus Biologicals) antibody was diluted 1:2000 ratio and blocked with 1% BSA in PBS buffer for 1 h at room temperature with rocking (50 rpm). 1 mL of blocked antibody was then incubated with fixed cells for 30 min on ice with rocking. Cells were washed with 1 mL of blocking buffer, pelleted by centrifugation at 500 rpm for 3 min and resuspended in 1 mL 0.1% BSA in PBS buffer and filtered through a cell strainer prior to analysis. Details of the gating strategies used for FACS are provided in the Supplementary Figures S4 and S5.

### Formation of ectopic tumors in mice and fluorescence in vivo imaging

Mice which lack a fully functional immune system, specifically 6 NOD-scid (NOD.CB17-Prkdc <scid>/J) mice at 9 weeks of age, were obtained from The Jackson Laboratories (ME, USA). All animal experiments were approved by the Institutional Animal Care and Use Committee (IACUC) of the University of Texas at Arlington and were conducted in accordance with the approved standards of humane animal care. The mice were fed standard chow and water *ad lib* and housed in the UT Arlington IACUC approved barrier facility under a 12 h light cycle. Three groups of mice (one male and one female in each group) were used for the growth of non-murine HEK293 background, BZ, and BZ-mCherry derived tumors. Specifically, after one week of acclimation to the barrier facility, the mice were anesthetized with 2% isoflurane followed by subcutaneous injection in the flank with 6 × 10^6^ cells per 100 μL PBS of either HEK293, BZ, or the BZ-mCherry cell line. The mice were returned to their cage and monitored weekly for tumor growth at the location of the injection. When a palpable tumor had formed, the animal was either euthanized to collect the tumor sample and surround tissue for further examination or instead anesthetized, shaved, and subjected to fluorescence in vivo imaging using a Perkin Elmer IVIS Lumina XRMS Series III after which the mouse was euthanized for tissue collection. Imaging was conducted with the following parameters: Emission = 620, Excitation = 580, Bin = 4/4, Fnumber = f2, exposure = 0.5 s.

### Harvesting of engrafted tumors and lysis or immunohistochemistry

After mice were euthanized by CO_2_ and cervical dislocation, a small incision was made on the abdomen and the skin separated to expose the underlying engrafted tumors. The tumor was excised, measured, and placed in either PBS for resuspension and lysis (as described above for glycosidase assay or immunoblotting) or placed in OCT for flash freezing and cryosectioning on polylysine slides at 5 μm using a cryotome with sections stored at − 80 °C. For immunohistochemistry, the sections were blocked overnight using 1% bovine serum albumin (BSA) in 10 mM PBS buffer. 1 μL of primary antibody (either anti-IgM heavy chain or anti-lambda light chain) was diluted with washing buffer (1% BSA and 0.5% Tween-20 in 10 mM PBS) to 1 mL and incubated overnight with slow horizontal agitation (50 rpm). The slide was washed with 10 mL of washing buffer for 10 min, three times with horizontal agitation. 1 μL of secondary antibody conjugated with HRP was diluted to 2 mL and incubated for 30 min at room temperature with agitation followed by three times washing. 10 mL of 0.05% AEC and 0.015% H_2_O_2_ in 50 mM acetate buffer pH 5.5 was added to develop the sections.

## Supplementary information

Supplementary Informations.

## Data Availability

All data generated or analysed during this study are included in this published article and through supplementary information.
