# Author Correction: Engineering a reporter cell line to mimic the high oligomannose presenting surface immunoglobulin of follicular lymphoma B cells

**DOI:** 10.1038/s41598-021-00032-z

**Published:** 2021-10-07

**Authors:** Butaek Lim, LeNaiya Kydd, Justyn Jaworski

**Affiliations:** Department of Bioengineering, University of Texas at Arlington, 500 UTA Blvd, Arlington, TX 76019 USA

Correction to: *Scientific Reports* 10.1038/s41598-020-79862-2, published online 8 January 2021

The original version of this Article contained an error in the Acknowledgments section.

"The authors would like to thank the University of Texas at Arlington for funds to support the research and animal studies conducted in this work. LeNaiya Kydd received support by the National Institutes of Health (NIH) training award, NIH T32 HL134613. Additional support was received by the National Institute of General Medical Sciences of the National Institute of Health under Award Number R15GM135892. The content is solely the responsibility of the authors and does not necessarily represent the official views of the National Institute of Health."

Now reads:

"The authors would like to thank the University of Texas at Arlington for funds to support the research and animal studies conducted in this work. LeNaiya Kydd received support by the National Institutes of Health (NIH) training award, NIH T32 HL134613. The content is solely the responsibility of the authors and does not necessarily represent the official views of the National Institute of Health."

