# Supplementary material for: Engineering a reporter cell line to mimic the high oligomannose presenting surface immunoglobulin of follicular lymphoma B cells
Source: Sci Rep. 2021 Jan 8;11:87. doi: 10.1038/s41598-020-79862-2 (PMC7794505; doi:10.1038/s41598-020-79862-2)
Supplement: Supplementary file 1 — Supplementary Informations. [file 41598_2020_79862_MOESM1_ESM.docx]

**Supplementary Information**

**Engineering a Reporter Cell Line to Mimic the High Oligomannose Presenting Surface Immunoglobulin of Follicular Lymphoma B Cells**

Butaek Lim^1^, LeNaiya Kydd^1^, and Justyn Jaworski^1*^

^1^Department of Bioengineering, University of Texas at Arlington, 500 UTA Blvd., Arlington, TX 76019, USA

**Corresponding Authors: J. Jaworski: Justyn.Jaworski@uta.edu*


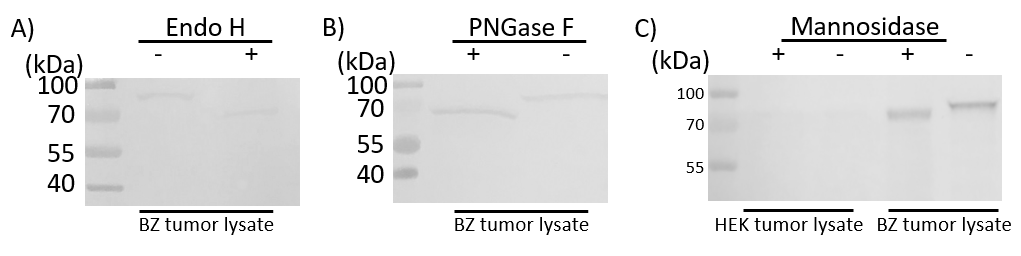


**Supplementary Figure 1 | Immunoblotting of glycosidase assay product from tumor lysate.** Tumor lysates from 4-5 week palpable tumors generated from BZ cells or HEK cells were used for glycosidase assays using (A) EndoH, (B) PNGase F, or (C) Mannosidase and separated by SDS-PAGE. Immunoblotting of the transfers and staining with anti-IgM heavy chain (primary) revealed a shift in the size of the heavy chain resulting from cleavage of the glycan. Only a very small band shift for cleavage by Mannosidase is seen as compared to EndoH or PNGase F. This was to be expected since the specificity of Mannosidase trims only the terminal mannose groups.


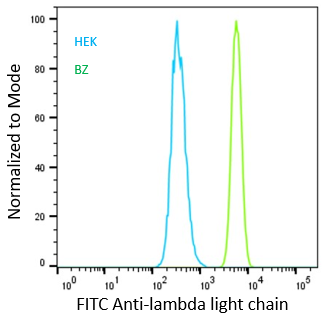


**Supplementary Figure 2 | FACS analysis of HEK and BZ cell lines after exposure to FITC labeled anti-lambda light chain probe.** The results show the BZ cells are positive for the display of antibody recognized by the FITC labeled anti-lambda light chain probe.


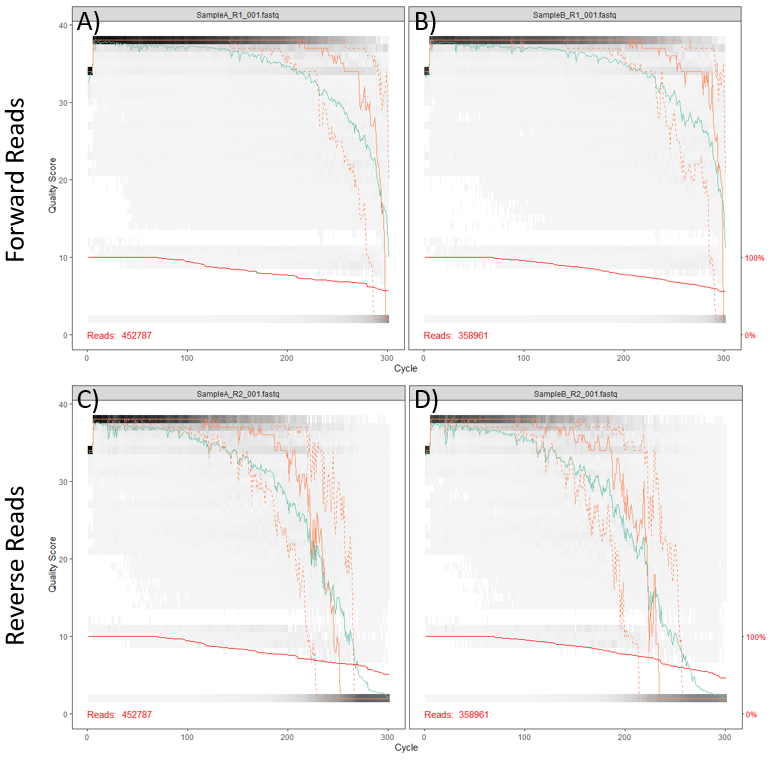


**Supplementary Figure 3 | Quality scores of NGS data.** Quality scores for the forward reads of (A) sample 1 and (B) sample 2 as well as for the reverse reads of (C) sample 1 and (D) sample 2. The gray scale provides a heat map of the frequency of the quality score at that particular base position (with dark colors corresponding to higher frequencies). The mean quality score is provided in green with respect to each base position. The orange line provides the median quality score distribution with the lower quartile 25^th^ percentile and upper quartile 75^th^ percentile in dashed orange lines. The red line shows the proportion of reads that extend at least to that base position.


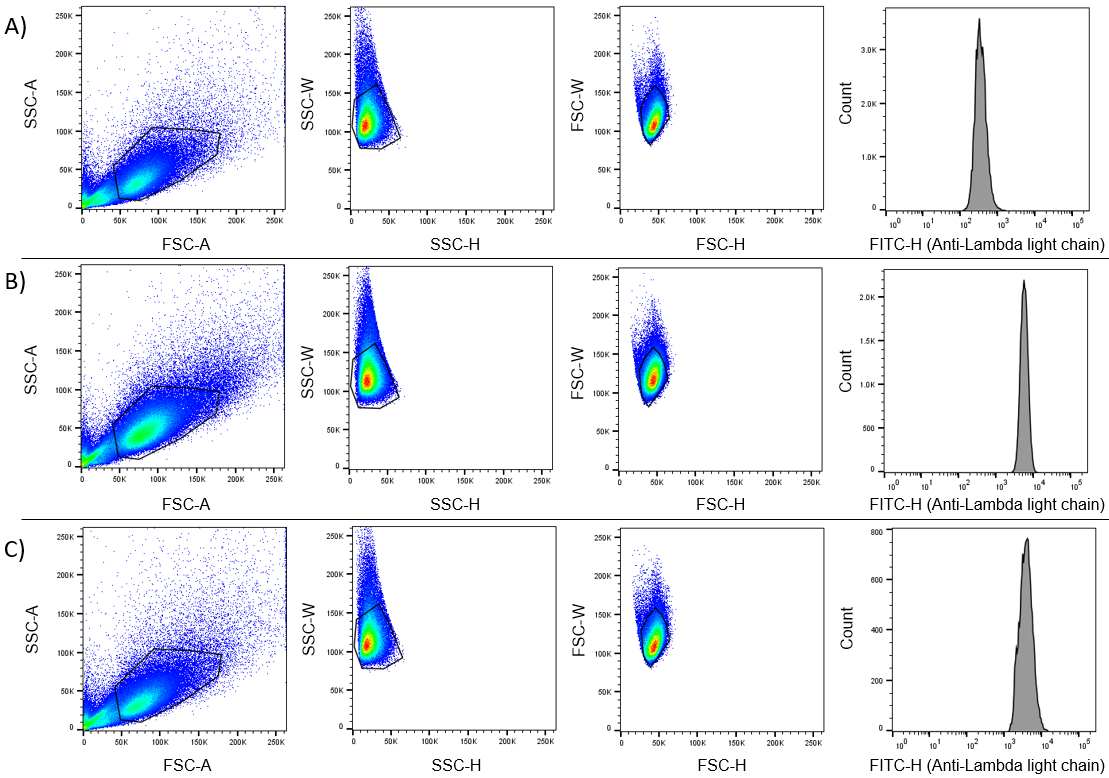


**Supplementary Figure 4 | Gating strategy for cells incubated with FITC labeled anti-lambda light chain probe.** Representative flow cytometry dots illustrating the gating strategy for examination of the presence of antibody on A) HEK293 cells, B) BZ cells, and C) BZ-mCherry cells. The results show the BZ and BZ-mCherry cells are positive for the display of antibody recognized by the FITC labeled anti-lambda light chain probe.


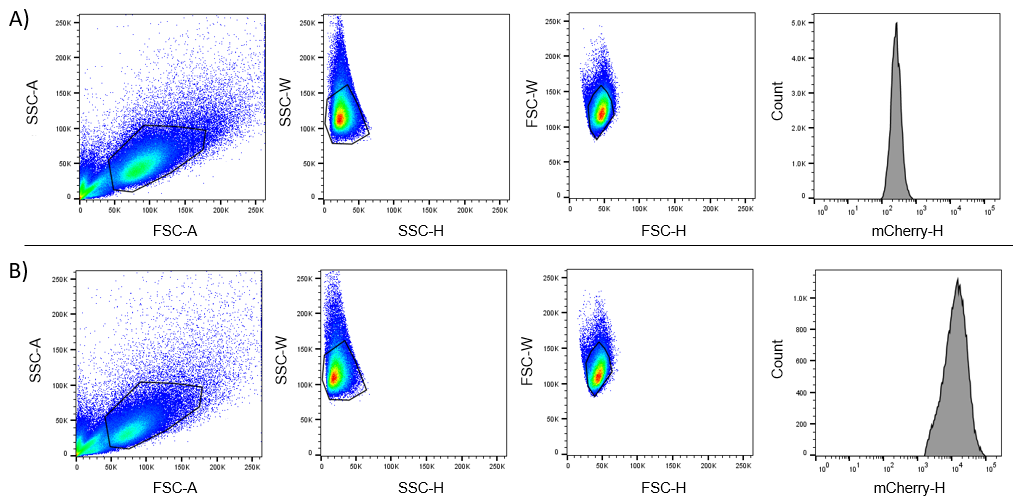


**Supplementary Figure 5 | Gating strategy for cells to examine fluorescence reporter.** Representative flow cytometry dots illustrating the gating strategy for examination of the presence of red fluorescence reporter within A) BZ cells and B) BZ-mCherry cells. The results show the BZ-mCherry cells as providing a high fluorescence from the expressed mCherry reporter.


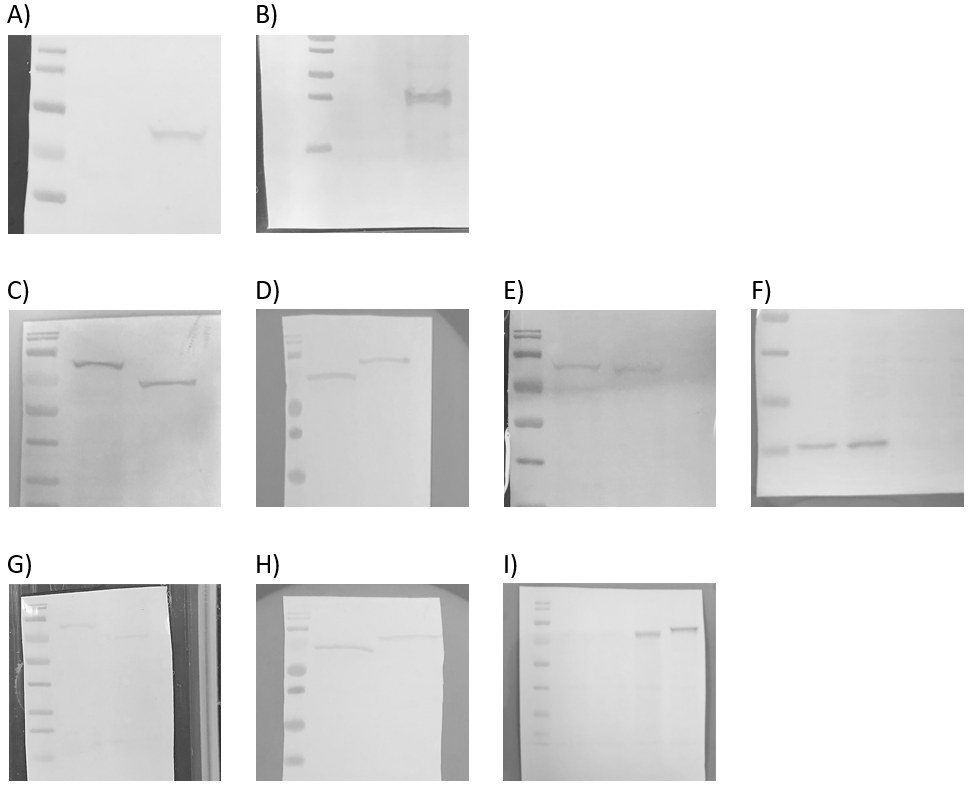


**Supplementary Figure 6| Uncropped photos taken of immunoblots for A) Figure 3a, B) Figure 3b, C) Figure 4a, D) Figure 4b, E) Figure 4c, F) Figure 4d, G) Supplementary Figure 1a, H) Supplementary Figure 1b, I) Supplementary Figure 1c.**


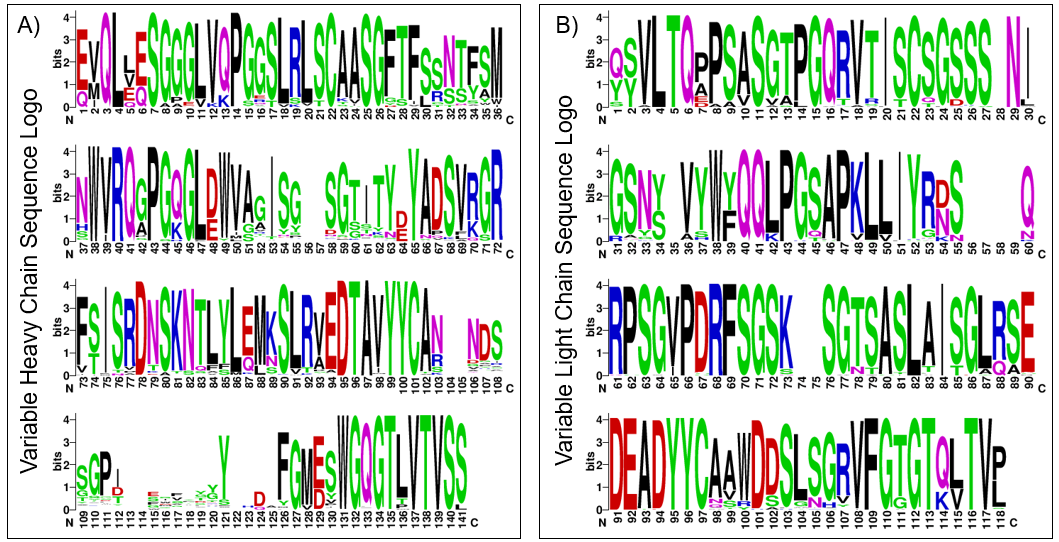


**Supplementary Figure 7 | Sequence logo of the variable heavy and lambda light chains.** Multiple sequence alignment of the amplicons analyzed and translated by IMGT/HighV-QUEST provided the consensus sequences of amino acids for (A) the variable heavy chain and (B) the variable lambda light chain where notably the N-D-S glycosylation site is seen in CDR3 of the variable heavy chain.


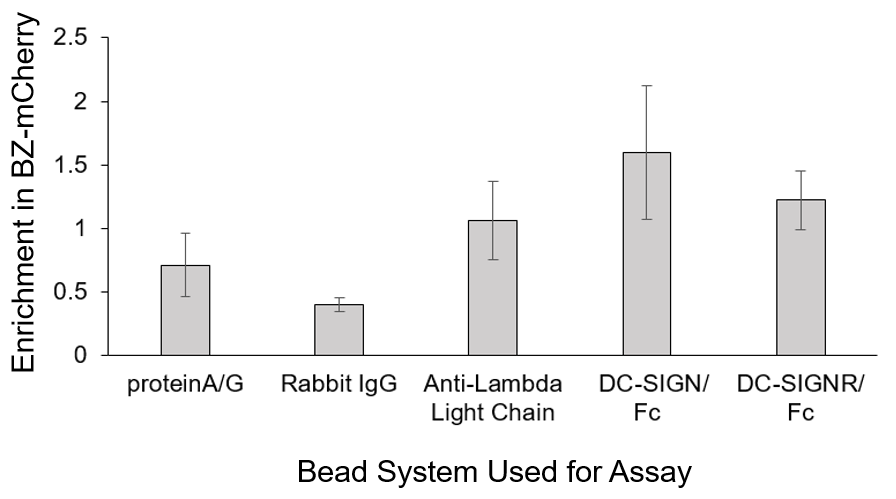


**Supplementary Figure 8 | Enrichment in BZ-mCherry reporter cells vs HEK293T cells using proteinA/G magnetic bead capture assay.** Significant enrichment of BZ-mCherry binding over the HEK293T background is observed for the proteinA/G beads labeled with C-lectin (DC-SIGN or DC-SIGNR) in comparison to the controls beads of unlabeled proteinA/G beads, proteinA/G beads labeled with purified rabbit IgG (negative control), and proteinA/G beads labeled with anti-lambda light chain (positive control).


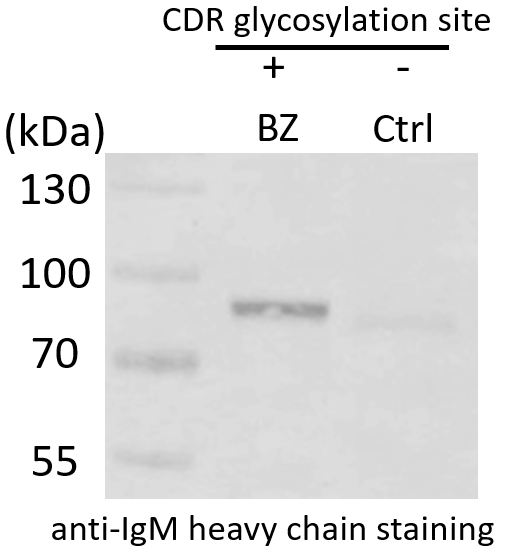


**Supplementary Figure 9 | Immunoblotting of cell lysates separated by SDS-PAGE of engineered BZ cell line and a negative control cell line lacking the CDR glycosylation site.** Anti-IgM heavy chain staining of the cell lysates of the BZ cell line as well as a negative control cell line possessing a mutated version of the heavy chain in which the N-D-S glycosylation site at CDR3 has been replaced by G-R-Y. The small band shift to a lower molecular weight of the negative control cell line is readily observable providing further evidence that the BZ cell line possesses a glycan at the N-D-S glycosylation site within the CDR3 of the heavy chain.

**Supplementary Methods:**

The following primers were used in preparation of amplicons for deep sequencing of Ig variable segments. (Note: R=A,G; W=A,T; S=C,G; D=A,G,T; B=C,G,T; N=A,C,G,T; Y=C,T; M=A,C; K=G,T; H=A,T,C; V=A,C,G)

**1st PCR extension**

Vlambda primers

IGLam V1for: ATC TAA CTC GAG CAG TCT GYS YTG ACK CAG CCK SC

IGLam V2for: ATC TAA CTC GAG AAT TTT ATG CTG ACT CAG CCC CA

IGLam V3for: ATC TAA CTC GAG TCY TMT GWG CTG ACT CAG SMM CC

IGLam Jrev: TAA ACT ATG CGG CCG CAC CTA RRA CGG TSA SCT KGG TCC C

Vkappa primers

IGKap V1for: ATC TAA CTC GAG GAA ACG ACA CTC ACG CAG TCT CC

IGKap V2for: ATC TAA CTC GAG GAY RTY GTG ATG ACY CAG TCT CC

IGKap V3for: ATC TAA CTC GAG GAA ATT GTG YTG ACK CAG TCT CC

IGKap V4for: ATC TAA CTC GAG GAC ATC CAG ATG ACC CAG TCT CC

IGKap Jrev: TAA ACT ATG CGG CCG CAC GTT TRA THT CCA SYY KKG TCC C

Vheavy primers

Vh1for: ATC TAA CTC GAG SAR RTV CAG CTS BWR SAG TCN GG

Vh2for: ATC TAA CTC GAG GAG GTG CAG CTG TTG CAG TCT GC

Vh3for: ATC TAA CTC GAG CAG GTA CAG CTG CAG CAG TCA GG

IGH Jrev: TAA ACT ATG CGG CCG CAC CTG ARG AGA CRG TGA CC

**2nd PCR extension**

Adapter primers

NGS Vhfor: (FPOA)-AATCTAACTCGAGSARRTVCAGC

NGS Vlambdafor: (FPOA)-TATCTAACTCGAGYMBTMTGHS

NGS Vkappafor: (FPOA)-GATCTAACTCGAGGAHRYBVHRHTYAC

NGS IGmix Jrev: (RPOA)-TAAACTATGCGGCCGCACSTD

Where FPOA is the Forward Primer Overhang Adapter (TCGTCGGCAGCGTCAGATGTGTATAAGAGACAG) and RPOA is the Reverse Primer Overhang Adapter (GTCTCGTGGGCTCGGAGATGTGTATAAGAGACAG).

**Detailed methods for deep sequencing of FFPE specimens from follicular lymphoma involved lymph nodes**

De-identified follicular lymphoma specimens were approved for use and purchased from US Biomax, Inc. (Rockville, MD, USA) tissue bank collection, which obtains specimens under high ethical standards with HIPPA protocols and informed consent of donors. These unstained FFPE slide specimens were of FL involved lymph nodes taken from the left groin of a 35 year old female. Specimens were first processed for DNA extraction by two 15 minute incubations in xylene followed by incubation in ethanol for subsequent extraction using the Qiagen FFPE DNA extraction kit. Deep sequencing of the variable heavy and light chain sequences presented in the samples were carried out by first generating amplicons using 20 cycles of PCR with the primer sets referred to as “1^st^ PCR Extension” which were based on existing primers optimized for generating libraries of the human Ig repertoire^1,2^. After amplicon size was confirmed by agarose gel electrophoresis and cleanup by column purification, a second fusion amplification of 5 cycles was performed using the primer sets referred to as “2^nd^ PCR extension” to append the amplicons with the Nextera adaptor sequences. Once again the amplicon size was assessed by gel and column purified before providing 58uL of sample DNA at 277ng/uL of dsDNA for NGS sequencing by MiSeq. Samples were processed and sequenced by Macrogen where first the Nextera XT indexing primers Index 1 Read (CAAGCAGAAGACGGCATACGAGAT[i7]GTCTCGTGGGCTCGG) and Index 2 Read (AATGATACGGCGACCACCGAGATCTACAC[i5]TCGTCGGCAGCGTC) were added where i7 and i5 are sets of distinct indexing sequences used as part of Macrogen’s Illumina MiSeq NGS workflow. The resulting sets of samples yielded 452787 and 358961 paired end (2x300nt read length) MiSeq reads of the NGS data obtained as FASTQ files.

The amplicon data was processed using Bioconductor v3.11 in R v4.0.0 with the DADA2 package v1.16.0 ^3^. The mean quality score at each base position was determined, and based on the quality profile standard filtering was conducted using truncation of the forward reads at position 250 and the reverse reads at position 180 in order to only keep the positions in which there was a high mean quality score distribution above 30. The parametric error model of the DADA2 algorithm was used to learn the error rates of our amplicon dataset and infer the true sequence variants while de-noising to remove spurious reads. The unique pairings were merged where paired reads that did not overlap were removed. Data processing workflow in R is provided below in these Supplementary Methods. The amplicons were then analyzed by IMGT/HighV-QUEST where they were translated to the amino acid sequences, individually aligned to their respective heavy or light chain sequences designate by subtype (heavy, kappa, lambda), and underwent mutational analysis with respect to the germ line sequence. Multiple sequence alignment of the translated sequences using MAFFT was provided via NGPhylogney.fr^4^. The consensus sequence of the most common amino acids was selected from the sequence logo provided by the aligned clonotypes as analyzed by WebLogo for both the heavy and light chain variable domains^5^. These consensus sequences for the light chain and heavy chain were then used to construct the antibodies to be displayed in our engineered cell line.

**Detailed methods for lectin-based bead assay**

In order to provide secondary validation of the high oligomannose displayed on our engineered cell line, we adapted a recently published lectin-based binding assay for the high mannose presented by follicular lymphoma B cells^6^. In brief, we carried out a competitive binding assay of the BZ-mCherry vs HEK control cells using magnetic beads labeled with the lectins DC-SIGN or DC-SIGNR that are known to interact with the high oligomannose presented by follicular lymphoma B cells. This assay was repeated with several sets of magnetic bead controls that were either unlabeled, labeled with anti-lambda light chain (positive control), or labeled with non-specific rabbit IgG (negative control). In detail, 10uL of 10mg/mL protein A/G magnetic beads (Pierce Thermo Scientific) were first mixed with 1mL of Tris buffer (containing 20mM Tris, 150mM NaCl, 2mM CaCl_2_, and 0.05% Tween-20) in microcentrifuge tubes and washed twice with this buffer by applying a neodymium magnet to collect the beads, discard the supernatant, and resuspending in fresh Tris buffer after removing the magnet. For preparation of lectin-labeled beads, 50uL of 0.1mg/mL DC-SIGN/Fc or DC-SIGNR/Fc (R&D Systems, Inc.) in 1 mL of Tris buffer containing 1%BSA was added to the washed beads and incubated for 1 hour with inversion at room temperature and blocked with 1%BSA overnight at 4^o^C. For preparation of rabbit IgG (negative control) beads or anti-lambda light chain (positive control) bead, 3uL of 5mg/mL rabbit IgG or 6uL of 1mg/mL anti-lambda light chain in 1 mL of Tris buffer containing 1%BSA was added to the washed beads and incubated for 1 hour with inversion at room temperature and blocked with 1%BSA overnight at 4^o^C. Unlabeled beads were also blocked with 1%BSA overnight at 4^o^C. All beads were washed and collected prior to the binding assay. BZ-mCherry cells and HEK293T cells were mixed in equal amounts at 5x10^5^ cells/tube in 1mL of Tris buffer containing 20ug of the respective labeled or unlabeled magnetic beads and incubated for 1 hour with slow rotation. The beads were then collected and unbound cells were removed. The sample was then washed twice with 1mL of Tris buffer and finally resuspended in 1mL of Tris buffer for analysis by flow cytometer to count the number of red fluorescent BZ-mCherry cells compared to non-fluorescent HEK293T cells. The experiments were performed in triplicate to obtain the average enrichment in BZ-mCherry relative to HEK293T.

**Detailed cloning methods**

In pFUSE for the heavy chain, codons for the consensus sequence of the variable heavy chain from the FL involved node (referred to here as VH3-23 and seen in Figure 2) was cloned into a pUC19 vector. To utilize the constant region of the IgM heavy chain, the CHIg-mM segment of pFUSE-CHIg-mM vector was amplified with forward (F-EcoRI-NheI-CHIg) and reverse (R-XbaI-KpnI-CHIg) primers and cloned into pUC19 vector. After amplifying VH3-23 sequence using forward (F-EcoRI-VH3-23) and reverse (R-NheI-VH3-23) primers, it was cloned and placed in the upstream of CHIg-mM to construct VH3-23-CHIg-mM. A transmembrane domain (TMD) was subcloned into the 3’ end of CHIg-mM after amplifying with forward (F-KpnI-TMD) and reverse (R-XbaI-TMD) primers and VH3-23-CHIg-mM-TMD was successfully constructed in pUC19 vector. The whole insert then cut with EcoRI and XbaI was transferred to the pFUSE backbone vector to construct pFUSE-VH3-23-CHIg-mM-TMD vector. Light chain cloning was conducted after amplifying the consensus variable lambda light chain (referred to here as VL1-47 and seen in Figure 2) by using forward (F-VL1-47) and reverse (R-VL1-47) primer and directly introduced into the pFUSE2ss-CLIg-mL1 vector to form pFUSE2ss-VH1-47-CLIg-mL1.

For site directed mutagenesis to replace the N-D-S glycosylation site for generating a negative control cell line, an inverse PCR approach was carried out on the pUC19 vector containing the VH3-23-CHIg-mM-TMD construct listed above. Specifically, the N-D-S sequence was replaced with G-R-Y in the CDR3 by carrying out inverse PCR of the VH3-23-CHIg-mM-TMD containing pUC19 plasmid with the primers (Ctrl-iPCR-for) and (Ctrl-iPCR-rev) followed by DpnI digest to remove the template strand and column purified. The DNA product was then cut with NotI and column purified followed by ligation using T4 DNA ligase and transformed into DH5alpha chemically competent cells. After sequence confirmation of the mutant clone possessing no CDR3 glycosylation site, the cells were inoculated in LB for overnight growth, the plasmid was column purified, and the insert was cut with EcoRI and XbaI followed by gel purification. The insert was then ligated with an EcoRI and XbaI cut pFUSE backbone vector to construct the Negative-Ctrl-pFUSE-VH3-23-CHIg-mM-TMD vector after transformation in DH5alpha chemically competent cells. After sequence confirmation, the Negative-Ctrl-pFUSE-VH3-23-CHIg-mM-TMD vector and pFUSE2ss-VH1-47-CLIg-mL1 were mixed in equimolar amounts at a 3:1 ratio with polyethylene imine for transfection of HEK293T cells and GFP-HEK293T cells (which had previously been transfected with the pEGFP-C1vector and selected to stably exhibit green fluorescence).

**Cloning and mutagenesis primers**

F-EcoRI-NheI-CHIg: AAAGGAATTCCCTAGCTAGCTCAGAGAGTCAGTCCTTCCC

R-XbaI-KpnI-CHIg: ATGCTCTAGAGCGGTACCAGTGGACTTGTCCACGGTCCTC

F-EcoRI-VH3-23: AAAGGAATTCCGAAGTACAGCTCTTAGAGTCGG

R-NheI-VH3-23: AACTAGCTAGCTGAGGAGACAGTGACCAGG

F-KpnI-TMD: ATTGAGGGTACCGAAGGAGAAGTGAACG

R-XbaI-TMD: TGTTTTCTAGATCACTTAACCTTGAACAAGGTAACAG

F-VL1-47: ATGGAATTCCAGTCCTATGTGCTGACTCAGGACCC

R-VL1-47: TATCAGGTACCTGTCCCGAATACACGGCCACTCAGGCTGTCATCCCATGCTGC

Ctrl-iPCR-for: ATAATAGCGGCCGCTATGGTGGCCCGATTTACTTTGAATCCTG

Ctrl-iPCR-rev: TAAGTTCATAGCGGCCGCTCGCACAGTAATATACGGCCGTGTC

**R code for processing NGS data**

> path<-"C:\\NGSdata"

> list.files(path)

> fnFs<-sort(list.files(path,pattern="_R1_001.fastq",full.names=TRUE))

> fnRs<-sort(list.files(path,pattern="_R2_001.fastq",full.names=TRUE))

> sample.names<-sapply(strsplit(basename(fnFs),"_"), `[`,1)

> plotQualityProfile(fnFs[1:2])

> plotQualityProfile(fnRs[1:2])

> filtFs <- file.path(path, "filtered", paste0(sample.names, "_F_filt.fastq.gz"))

> filtRs <- file.path(path, "filtered", paste0(sample.names, "_R_filt.fastq.gz"))

> names(filtFs) <- sample.names

> names(filtRs) <- sample.names

> out <- filterAndTrim(fnFs, filtFs, fnRs, filtRs, truncLen=c(250,180), maxN=0, maxEE=c(2,2), truncQ=2, rm.phix=TRUE, compress=TRUE, multithread=FALSE)

> head(out)

> errF<-learnError(filtFs, multithread=FALSE)

> errF<-learnErrors(filtFs, multithread=FALSE)

> plotErrors(errF, nominalQ=TRUE)

> dadaFs<-dada(filtFs, err=errF, multithread= FALSE)

> dadaFs[[1]]

> errR<-learnErrors(filtRs, multithread=FALSE)

> plotErrors(errR, nominalQ=TRUE)

> dadaRs<-dada(filtRs, err=errR, multithread= FALSE)

> mergers<-mergePairs(dadaFs, filtFs, dadaRs, filtRs, verbose=TRUE)

> write.table(mergers[[2]], file="smp2.txt", sep=",",quote=FALSE, row.names =F)

> write.table(mergers[[1]], file="smp1.txt", sep=",",quote=FALSE, row.names =F)

> seqtab<-makeSequenceTable(mergers)

> dim(seqtab)

> table(nchar(getSequences(seqtab)))

**Supplementary References**

1 Marks, J. D. *et al.* By-passing immunization: human antibodies from V-gene libraries displayed on phage. *Journal of molecular biology* **222**, 581-597 (1991).

2 Marks, J. D., Tristem, M., Karpas, A. & Winter, G. Oligonucleotide primers for polymerase chain reaction amplification of human immunoglobulin variable genes and design of family‐specific oligonucleotide probes. *European journal of immunology* **21**, 985-991 (1991).

3 Callahan, B. J. *et al.* DADA2: High-resolution sample inference from Illumina amplicon data. *Nature Methods* **13**, 581-583, doi:10.1038/nmeth.3869 (2016).

4 Katoh, K. & Standley, D. M. MAFFT multiple sequence alignment software version 7: improvements in performance and usability. *Molecular biology and evolution* **30**, 772-780 (2013).

5 Crooks, G. E., Hon, G., Chandonia, J.-M. & Brenner, S. E. WebLogo: a sequence logo generator. *Genome research* **14**, 1188-1190 (2004).

6 Amin, R. *et al.* DC-SIGN–expressing macrophages trigger activation of mannosylated IgM B-cell receptor in follicular lymphoma. *Blood, The Journal of the American Society of Hematology* **126**, 1911-1920 (2015).
